# Supplementary material for: Highly sensitive quantitative phase microscopy and deep learning aided with whole genome sequencing for rapid detection of infection and antimicrobial resistance
Source: Front Microbiol. 2023 Apr 12;14:1154620. doi: 10.3389/fmicb.2023.1154620 (PMC10130531; doi:10.3389/fmicb.2023.1154620)
Supplement: Supplementary file 1 [file Data_Sheet_1.docx]

Supplementary Material

**Highly sensitive quantitative phase microscopy and deep learning complement whole genome sequencing for rapid detection of infection and antimicrobial resistance**

**Azeem Ahmad^1^, Ramith Hettiarachchi^2,3,§^, Abdolrahman Khezri^4,§^, Balpreet Singh Ahluwalia^1,5,#^, Dushan N.Wadduwage^3,#^, Rafi Ahmad^4,6,#,*^**

*^1^Department of Physics and Technology, UiT The Arctic University of Norway, Tromsø, 9037, Norway.*

*^2^Department of Electronic and Telecommunication Engineering, University of Moratuwa, Sri Lanka*

*^3^Center for Advanced Imaging, Faculty of Arts and Sciences, Harvard University, Cambridge, USA.*

*^4^Department of Biotechnology, Inland Norway University of Applied Sciences, Holsetgata 22, 2317, Hamar, Norway.*

*^5^Department of Clinical Science, Intervention and Technology, Karolinska Insitute, 17177 Stockholm, Sweden.*

*^6^Institute of Clinical Medicine, Faculty of Health Sciences, UiT - The Arctic University of Norway, 9037, Norway.*

Corresponding author: [rafi.ahmad@inn.no](mailto:rafi.ahmad@inn.no)

^§^These authors contributed equally.

^#^These authors contributed equally.

# Supplementary data

### Phase recovery algorithm

Quantitative phase microscopy system works on the principle of interferometry and generates 2D intensity modulated signal also called interferograms. For phase recovery of bacteria samples, multiple phase shifted interferograms ($H_{n}$) are utilized, which mathematically can be represented as follows (1, 2):

| $H_{n}\left( x,y \right)=a_{n}\left( x,y \right)+b_{n}\left( x,y \right)cos[\phi\left( x,y \right)+\delta_{n}]$ | (1) |
| --- | --- |

where $a_{r}\left( x,y \right)$ and $b_{r}(x,y)$ represent background and modulation amplitude of the interferogram, respectively. The subscript n represents the n^th^ phase shifted modulated intensity pattern (n = 1,2,3,…,N) with phase shift $\delta_{n}$ between the consecutive interferograms. N is the number of phase shifted frames. $\phi\left( x,y \right)$ is the spatial phase map related to the specimen. Eq. (1) can be written as follows:

| $H_{n}\left( x,y \right)=a_{n}\left( x,y \right)+b_{n}\left( x,y \right)\left[ \cos\left( \phi\left( x,y \right) \right)\cos{(\delta}_{n})-\sin\left( \phi\left( x,y \right) \right)\sin(\delta_{n}) \right]$ | (2) |
| --- | --- |

If the background $a_{n}\left( x,y \right)$ and the modulation $b_{n}\left( x,y \right)$ terms don’t variate in all the phase shifted interferograms, then Eq. (2) can be expressed as:

| $H_{n}\left( x,y \right)=A\left( x,y \right)+B\left( x,y \right)\left[ \cos\left( \phi\left( x,y \right) \right)\cos{(\delta}_{n})-\sin\left( \phi\left( x,y \right) \right)\sin(\delta_{n}) \right]$ | (3) |
| --- | --- |

With the background subtracted interferograms, Eq. (3) can be written in the simplified form as follows:

| $H_{n}\left( x,y \right)=\alpha_{n}H_{c}+\beta_{n}H_{s}$ | (4) |
| --- | --- |

where

$$\alpha_{n}=\cos{(\delta}_{n}); \beta_{n}=\sin(\delta_{n}); (5)$$

$$H_{c}(x,y)=B\left( x,y \right)\cos\left( \phi\left( x,y \right) \right) (6)$$

$$H_{s}(x,y)=B\left( x,y \right)\sin\left( \phi\left( x,y \right) \right) (7)$$

It can be seen from Eq. (4), any interferograms can be decomposed into two orthogonal signals $H_{c}(x,y)$ and $H_{s}(x,y)$.

Using principal component analysis (PCA), N phase shifted interferograms can be represented by smallest number of uncorrelated interferograms called the principal components. The first two principal components with largest eigen values correspond to the uncorrelated signals $H_{c}(x,y)$ and $H_{s}(x,y)$. The detailed mathematical steps to find two uncorrelated signals from N phase shifted interferograms can be found in Ref. (1). Then the spatial phase map of the sample can be obtained by employing Eq. (6) and Eq. (7) and represented by the following relation

| $\phi\left( x,y \right)={tan}^{-1}\left[ \frac{H_{s}(x,y)}{H_{c}(x,y)} \right].$ | (8) |
| --- | --- |

The amplitude map of the sample can be obtained using the following expression

| $E_{0}\left( x,y \right)=\sqrt{H_{s}^{2}\left( x,y \right)+H_{c}^{2}\left( x,y \right)}$ | (9) |
| --- | --- |

Using Eqs. (8) and (9), the complex field information of the specimen can be obtained as follows:

| $E\left( x,y \right)=E_{0}\left( x,y \right)exp(j\phi\left( x,y \right))$ | (10) |
| --- | --- |

The obtained complex field information is further utilized for the correction of the defocus amount from the reconstructed spatial phase maps. Defocus correction is necessary to avoid any error in the reconstructed phase maps, otherwise it significantly affects the reconstruction. The defocus correction is done by implementing angular spectrum method (3, 4) and its mathematical steps are provided in the next section.

**Defocus correction algorithm**

For accurate quantification of the phase maps of the bacteria samples, a defocus correction algorithm based on the angular spectrum propagation method is implemented. The complex signal (Eq. (10)) of the sample obtained from PCA algorithm is utilized to calculate spatial frequency spectrum by taking its Fourier transform. Mathematically, it can be represented as follows (4):

$$E\left( f_{x},f_{y};0 \right)=\iint_{-\propto}^{\propto} E(x,y;0)e^{-j2\pi(f_{x}x+f_{y}y)}dxdy (11)$$

where, $f_{x}=\alpha/\lambda$ and $f_{y}=\beta/\lambda$ represent the spatial frequencies of the wave vector along x and y direction. $\alpha$ and $\beta$ are the direction cosines of the wave vector with respect to x and y axis and $\lambda$ is the wavelength of light.

The obtained angular spectrum $E\left( f_{x},f_{y};0 \right)$ is further propagated along the direction of wave field propagation (z-axis) by different distances ‘z1’ to obtain the distribution of spatial frequency spectrum at different planes. This is obtained by employing the following expression (3, 4):

$$E\left( f_{x},f_{y};z_{1} \right)=E\left( f_{x},f_{y};0 \right)H(f_{x},f_{y}) \left( 12 \right)$$

where, $H(f_{x},f_{y})$ is the freespace optical transfer function and mathematically written as follows []:

$$H\left( f_{x},f_{y} \right)=\left\{ \begin{matrix} e^{-j\frac{2\pi z_{1}}{\lambda}\sqrt{1-\left( {\lambda f}_{x} \right)^{2}-{{\lambda f}_{y}}^{2}}}, & \sqrt{f_{x}^{2}+f_{y}^{2}}<\frac{1}{\lambda} \\ 0, & \mathrm{otherwise} \end{matrix} \right. (13)$$

The inverse Fourier transform of Eq. (12) provides the information of complex field information of the specimen at different planes ‘$E\left( x,y;z_{1} \right)$’, which is expressed as follows:

$$E\left( x,y;z_{1} \right)=\iint_{-\propto}^{\propto} E(f_{x},f_{y};z_{1})e^{j2\pi(f_{x}x+f_{y}y)}df_{x}df_{y} (14)$$

With known defocus distance ‘say d’, the complex field information thus obtained can be further utilized for the accurate quantification of phase information of the specimens.

To calculate the amount of defocus in the recovered phase maps, the complex field obtained in Eq. (10), is propagated from – z to + z in a step of Δz. The amplitude variance ‘$M_{\mathrm{var}}^{d}$’ corresponding to the complexfields at each propagation distance is then calculated and plotted as a function of the propagation distance called sharpness curve. Mathematically, the amplitude variance is written as follows (3, 5):

$$M_{\mathrm{var}}^{z_{1}}=\frac{1}{\mu}\sum\sum\left[ \left| E\left( x,y;z_{1} \right) \right|-\mu\right]^{2} (15)$$

where μ is the average of the amplitude of the complex field ‘$E\left( x,y;z_{1} \right)$’. The position corresponding to the minimum value of amplitude variance (say d) in the sharpness curve represents the amount of defocus present in the recorded phase data. The complex field information of the specimen is then propagated by distance ‘d’ using Eq. (14) to obtain the defocus corrected phase maps.

# Supplementary Figures and Tables

## Supplementary Figures


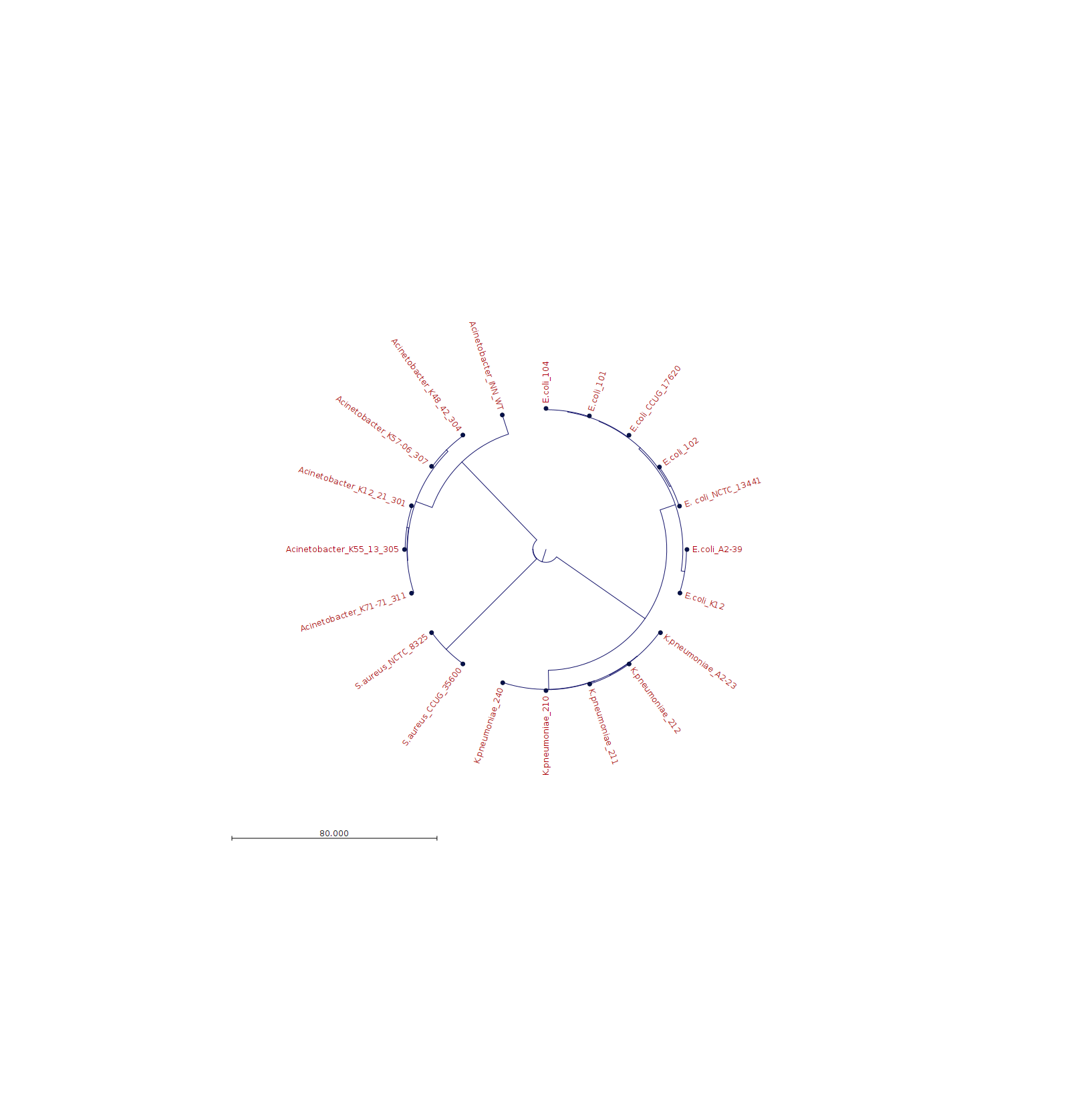


**Supplementary Figure 1:** Neighbour-joining phylogenetic tree based on average nucleotide identity data (obtained from whole genome alignment). The scale bar indicates the proportion of site changes along each branch.

**Supplementary Figure 2**: The loss value curves of the trained models: A) Antibiotic resistant/sensitive prediction, B) Gram stain classification, C) Species-level classification, and D) Strain classification.

Supplementary Figure 2 shows the training and validation loss curves of the four models trained for each classification task. These models are trained to minimize the cross-entropy loss.

## Supplementary Tables

**Table S1:** An overview of basic statistics for assembled genomes. Parameters with orange, blue, and green backgrounds are reported by BUSCO, QUAST, and Prokka tools, respectively. bp; base pair, Com; completed (both single and duplicated copies), Frag; fragmented, Miss; missed genes.

|  | **Com**  **(%)** | **Frag**  **(%)** | **Miss**  **(%)** | **Number of Contig** | **Total**  **Length (bp)** | **GC (%)** | **N50**  **(bp)** | **CDS** | **rRNA** | **tRNA** |
| --- | --- | --- | --- | --- | --- | --- | --- | --- | --- | --- |
| Acinetobacter_K12-21 | 99.7 | 0.1 | 0.2 | 104 | 4132096 | 39.13 | 144764 | 2152 | 3 | 44 |
| Acinetobacter_K48-42 | 99.5 | 0.4 | 0.1 | 95 | 3954176 | 39.11 | 92979 | 2140 | 3 | 42 |
| Acinetobacter_K55-13 | 99.7 | 0.1 | 0.2 | 136 | 3920963 | 39.03 | 76069 | 2098 | 3 | 46 |
| Acinetobacter_K57-06 | 99.5 | 0.4 | 0.1 | 112 | 3941154 | 38.94 | 81398 | 2157 | 3 | 63 |
| Acinetobacter_K71-71 | 99.6 | 0.3 | 0.1 | 116 | 4180919 | 39.08 | 90401 | 2168 | 3 | 63 |
| Acinetobacter_INN_WT | 99.6 | 0 | 0.4 | 153 | 3717898 | 40.17 | 52939 | 2057 | 5 | 34 |
| **Acinetobacter_Median** | **99.6** | **0.2** | **0.1** | **114** | **3947665** | **39** | **85900** | **2146** | **3** | **45** |
| E.coli_101 | 100 | 0 | 0 | 75 | 4952944 | 50.54 | 262450 | 3545 | 3 | 74 |
| E.coli_102 | 100 | 0 | 0 | 80 | 5072015 | 50.70 | 319576 | 3594 | 5 | 79 |
| E.coli_104 | 100 | 0 | 0 | 100 | 5052925 | 50.57 | 254457 | 3590 | 4 | 83 |
| E.coli_A2-39 | 100 | 0 | 0 | 269 | 5735389 | 50.42 | 102833 | 3749 | 5 | 89 |
| E.coli_NCTC_13441 | 3.4 | 10.5 | 86.1 | 61 | 4765744 | 51.05 | 116373 | 4036 | 12 | 61 |
| E. coli_CCUG_17620 | 100 | 0 | 0 | 1 | 5130767 | 50.54 | 5130767 | 3733 | 3 | 85 |
| E. coli_K12 | 100 | 0 | 0 | 1 | 4641652 | 50.79 | 4641652 | 3667 | 3 | 89 |
| **E.coli_Median** | **100** | **0** | **0** | **75** | **5052925** | **50.57** | **262450** | **3667** | **4** | **83** |
| K.pneumoniae_210 | 39.8 | 15.9 | 44.3 | 1451 | 2947026 | 58.15 | 2166 | 1716 | 4 | 13 |
| K.pneumoniae_211 | 98.9 | 0.2 | 0.9 | 82 | 5374307 | 57.35 | 251480 | 3709 | 3 | 78 |
| K.pneumoniae_212 | 86.6 | 4.5 | 8.9 | 905 | 4824002 | 58.22 | 7873 | 3223 | 2 | 42 |
| K.pneumoniae_240 | 98.9 | 0.2 | 0.9 | 84 | 5797728 | 56.67 | 200979 | 3851 | 4 | 79 |
| K.pneumoniae_A2-23 | 98.6 | 0.2 | 1.2 | 88 | 5737695 | 56.82 | 350054 | 3803 | 4 | 78 |
| **K.pneumoniae_Median** | **98.6** | **0.2** | **1.2** | **88** | **5374307** | **57.35** | **200979** | **3709** | **4** | **78** |
| S.aureus_CCUG_35600 | 40.7 | 32.9 | 26.4 | 40 | 3050840 | 33.09 | 2123257 | 2684 | 17 | 60 |
| S.aureus_NCTC_8325 | 99.3 | 0.7 | 0 | 1 | 2821361 | 32.87 | 2821361 | 1784 | 3 | 62 |
| **S.aureus_Median** | **70** | **16.8** | **13.2** | **20.5** | **2936101** | **32.98** | **2472309** | **2234** | **10** | **61** |


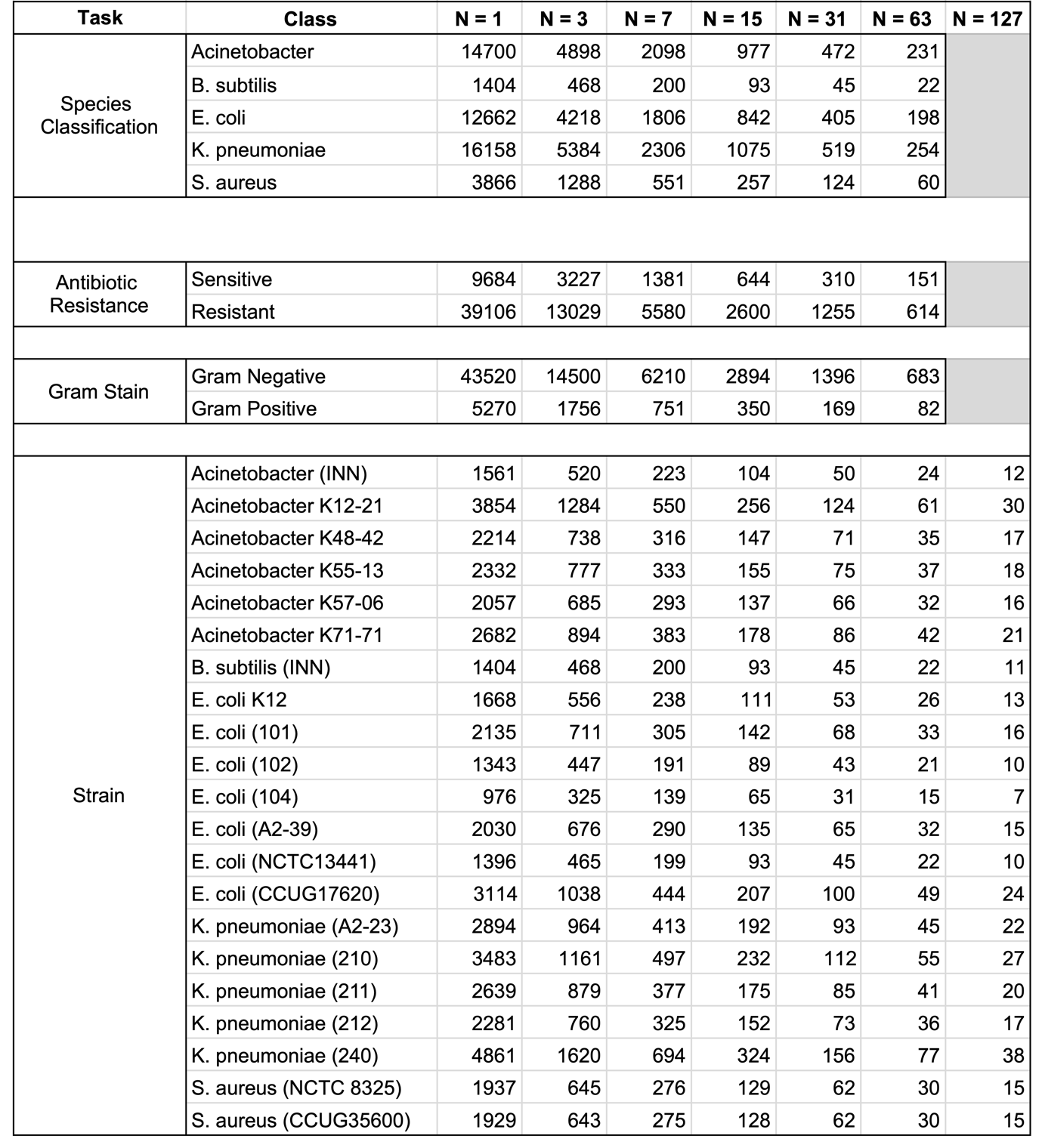


**Table S2:** Class distributions of the blind test set at each isolated number of bacteria (N) in the sample.


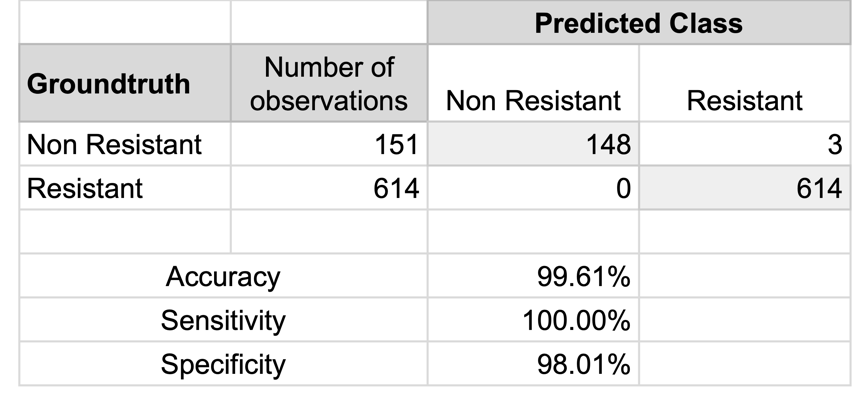


**Table S3:** Detailed classification metrics of antibiotic resistance at N = 63.


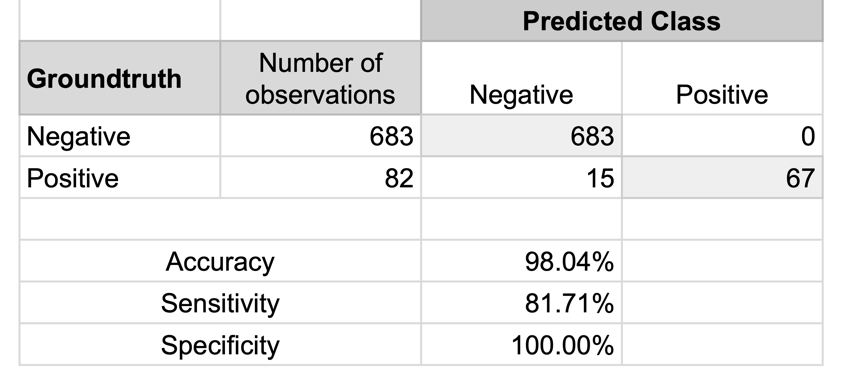


**Table S4:** Detailed classification metrics of gram stain classification at N = 63.


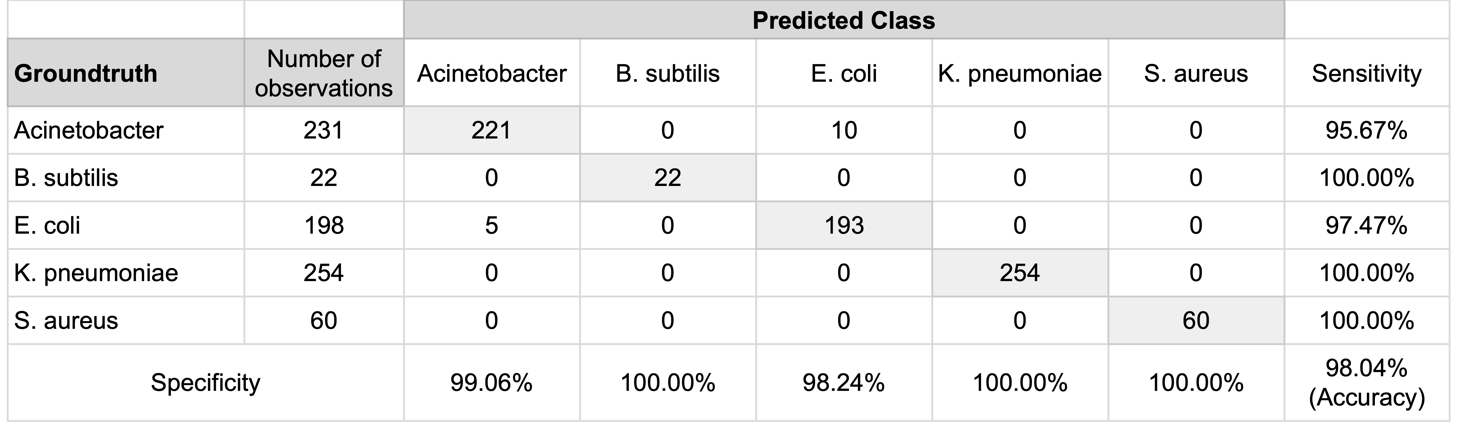
**Table S5:** Detailed classification metrics of species classification at N = 63.


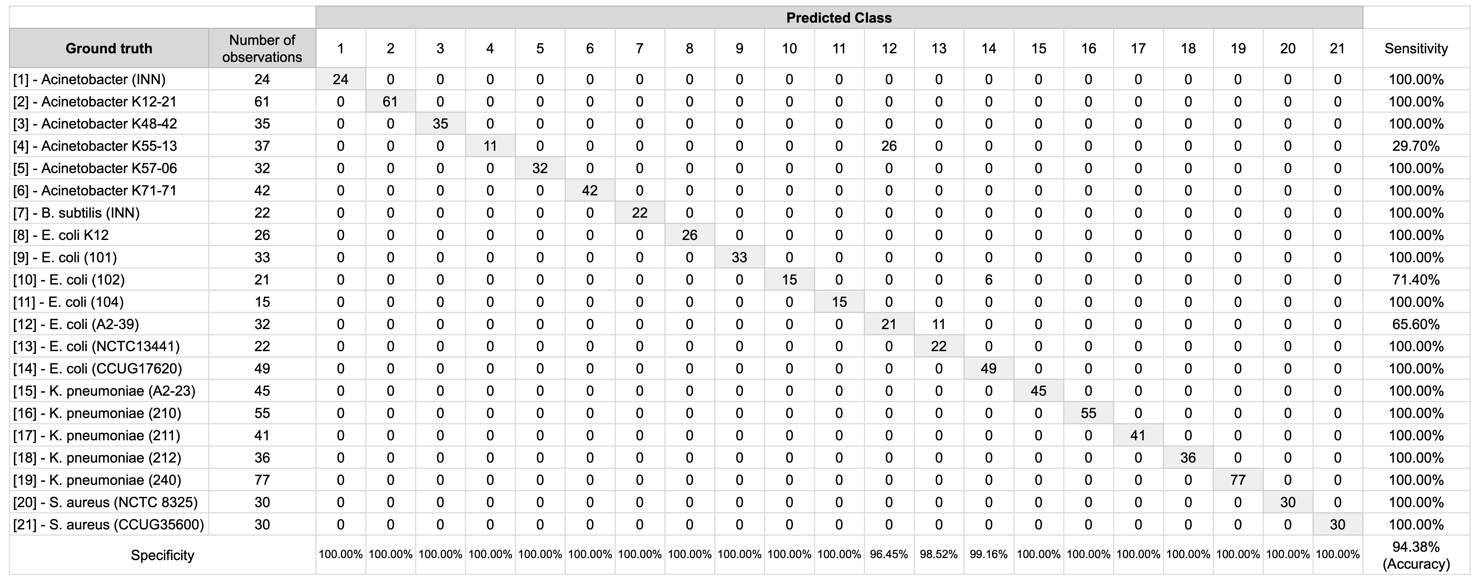
**Table S6:** Detailed classification metrics of strain classification at N = 63.

**References:**

1. J. Vargas, J. A. Quiroga, T. Belenguer, Phase-shifting interferometry based on principal component analysis. *Opt. Lett.* **36**, 1326-1328 (2011).

2. A. Ahmad *et al.*, Sub-nanometer height sensitivity by phase shifting interference microscopy under environmental fluctuations. *Opt. Express* **28**, 9340-9358 (2020).

3. A. Ahmad *et al.*, Quantitative phase microscopy of red blood cells during planar trapping and propulsion. *Lab Chip* **18**, 3025-3036 (2018).

4. M. T. Rinehart, H. S. Park, A. Wax, Influence of defocus on quantitative analysis of microscopic objects and individual cells with digital holography. *Biomed. Opt. Express* **6**, 2067-2075 (2015).

5. M. Doğar, H. A. İlhan, M. Özcan, Real-time, auto-focusing digital holographic microscope using graphics processors. *Rev. Sci. Instrum.* **84**, 083704 (2013).
